# Correlation between Acylcarnitine and Peripheral Neuropathy in Type 2 Diabetes Mellitus

**DOI:** 10.1155/2022/8115173

**Published:** 2022-02-17

**Authors:** Zhenni An, Danmeng Zheng, Dongzhuo Wei, Dingwen Jiang, Xuejiao Xing, Chang Liu

**Affiliations:** ^1^Department of Endocrinology, First Affiliated Hospital of Jinzhou Medical University, 121001, China; ^2^Department of Clinical Discipline of Chinese and Western Integrative Medicine, Liaoning University of Traditional Chinese Medicine, 110847, China

## Abstract

**Objective:**

In patients with type 2 diabetes mellitus (T2DM), it is unknown whether acylcarnitine changes in the patient's plasma as diabetic peripheral neuropathy (DPN) occurs. The purpose of the present study was to investigate the correlation between acylcarnitines and DPN in Chinese patients with T2DM.

**Methods:**

A total of 508 patients admitted to the First Affiliated Hospital of Jinzhou Medical University were included in this study, and all of whom were hospitalized for T2DM from January 2018 to December 2020. The diagnostic criteria for DPN were based on the 2017 Chinese Guidelines for the Prevention of Type 2 Diabetes. The contents of 25 acylcarnitine metabolites in fasting blood were determined by mass spectrometry. The measured acylcarnitines were classified by factor analysis, and the factors were extracted. To determine the correlation between acylcarnitines and DPN, binary logistic regression analysis was applied.

**Results:**

Among the 508 T2DM patients, 270 had DPN. Six factors were extracted from 25 acylcarnitines, and the cumulative contribution rate of variance was 61.02%. After the adjustment for other potential confounding factors, such as other carnitines and conventional risk factors, Factor 2 was positively associated with an increased risk of DPN (OR: 1.38, 95% CI: 1.13-1.69). Factor 2 contained acetylcarnitine (C2), propionylcarnitine (C3), butylcarnitine (C4), and isovalerylcarnitine (C5).

**Conclusions:**

Plasma levels of short-chain acylcarnitines (C2, C3, C4, and C5) were positively associated with DPN risk.

## 1. Introduction

According to statistics, the global incidence of diabetes among adults was 8.4% in 2017 and will rise to 9.9% by 2045. In addition, the incidence of diabetic complications has increased accordingly [1]. As a common complication of diabetes, DPN affects approximately half of the patients with diabetes. Pain and paraesthesia caused by DPN contribute to a serious decrease in the quality of life for diabetic patients [2]. Based on genomics and proteomics, metabolomics further explores life activities from the level of metabolites [3] and provides new ideas for the pathogenesis, diagnosis, and drug intervention of diseases by discovering potential biomarkers and metabolic pathways [4]. Acylcarnitine is an esterified L-carnitine and fatty acid that regulates glycolipid metabolic balance by participating in the *β*-oxidation of fatty acids [5]. Although glucose metabolism has been the focus of research on diabetes and its complications, the role of lipid metabolism in the occurrence and development of diabetes has also received increasing attention [6]. Previous studies have shown that plasma acylcarnitines and acylcarnitine levels in newly diagnosed T2DM patients are higher than those in non-T2DM patients [7, 8]. There are few clinical studies on the correlation between DPN and acylcarnitine, and the correlation between DPN and acylcarnitines has not been determined. Therefore, based on a cross-sectional approach, we investigated the relationship between plasma acylcarnitine metabolites and DPN in hospitalized Chinese patients with T2DM.

## 2. Materials and Methods

### 2.1. Methods and Population

A total of 508 patients with T2DM admitted to the First Affiliated Hospital of Jinzhou Medical University from January 2018 to December 2020 were included for a cross-sectional study to explore the relationship between blood acylcarnitines and DPN in T2DM patients. The basic patient information and metabolite data required by the study were searched by using the electronic medical record system of the First Affiliated Hospital of Jinzhou Medical University, China. The case inclusion criteria were as follows: (1) all patients were 18 years old or older; (2) according to the 1999 World Health Organization diagnostic criteria for type 2 diabetes, the diagnosis of T2DM was clear; and (3) the diagnosis of DPN conformed to the diagnostic criteria in the 2017 Guidelines for the Prevention and Treatment of Type 2 Diabetes in China. The case exclusion criteria were as follows: (1) younger than 18 years old; (2) type 1 diabetes mellitus, gestational diabetes mellitus (GDM), and other special diabetes mellitus; (3) acute complications of diabetes, such as diabetic ketoacidosis and hyperosmolar hyperglycaemia syndrome; (4) critical conditions, such as severe heart disease, severe lung disease, liver dysfunction, kidney dysfunction, tumour, acute infection, gangrene, and amputation; (5) neuropathy caused by other factors, such as alcohol, nutrition, family, uraemia, drugs, toxicants, and other factors; and (6) pregnancy.

The study was approved by the Clinical Research Ethics Committee of the First Affiliated Hospital of Jinzhou Medical University, and informed consent was waived by the Clinical Research Ethics Committee of the First Affiliated Hospital of Jinzhou Medical University due to the retrospective nature of the cross-sectional study.

### 2.2. Data Collection

An elevated weight metre was used to record the height and weight of the patients. Body mass index (BMI) was calculated by dividing weight (kg) by height (m^2^) squared. According to the recommendation of the Chinese Diabetes Association, BMI ≥ 24.0 kg/m^2^ but <28.0 kg/m^2^ is considered overweight, and BMI ≥ 28.0 kg/m^2^ is considered obese [9]. After resting in a quiet environment for 10 minutes, the patient sat and measured the blood pressure of the right upper limb with a standard mercury sphygmomanometer and cuff compression method. The patients fasted for at least 8 hours. Venous blood was collected, and cholesterol (TC), triglyceride (TG), low-density lipoprotein cholesterol (LDL-C), and high-density lipoprotein cholesterol (HDL-C) were detected by an Abbott automatic biochemical analyser. Glycated haemoglobin (HbA1c) was detected by borate affinity chromatography and an automatic HbA1c analyser. C peptide (CP) was determined using the Abbott immunobiochemical pipeline (chemiluminescence method). The motor nerve conduction velocity and sensory nerve conduction velocity of the patients were measured with a Medtronic Keypoint table electromyography and evoked potentiometer. The indoor temperature and skin temperature were kept at 21-25°Cand 25-32°C, respectively, during detection.

### 2.3. Acylcarnitine Quantification

Three drops of the subject's venous blood were collected, placed on a special filter paper, and dried naturally at room temperature. The filter paper was punched, and the disc was placed in a 96-well plate. Then, 100 *μ*l of internal standard was added to the 96-well plate, which was shaken and then centrifuged (2000 rpm, 2 min), and the filtrate was collected and added to another 96-well plate. Four different concentrations of amino acid and carnitine quality control solutions were added to four blank wells in a 96-well plate. Subsequently, the samples were subjected to nitrogen blowing at a temperature of 55°C for 8-10 minutes. After turning on the film press machine (180°C), 60 *μ*l of derivatization agent was added, and the film was pressed for 4 s followed by incubation for 20 minutes. The film was then removed, and the samples were subjected to nitrogen blowing for 15-20 min for drying. The samples were reconstituted, and the plate was wrapped in aluminium foil before analysis [10]. The MS metabolomic analysis was performed using the AB Sciex 4000 QTRAP System (AB Sciex, Framingham, MA, USA). The electrospray ionization source was an ion source, and the ion spraying voltage was 4.5 kV. The scanning mode of the analyte was used, and the mobile phase load test component was an 80% acetonitrile solution. The Cambridge Laboratory (Tewkesbury, Massachusetts, USA) isotope-labelled acylcarnitine absolute quantitative internal standard was used [10, 11].

### 2.4. Statistical Analysis

All statistical steps were performed using SPSS 23.0 software. The results were considered statistically significant when *P* < 0.05. First, we determined whether the variable was continuous. The normal distribution test was performed for the continuous variables, using the PP graph or QQ graph in this process. When the variables conformed to a normal distribution, the mean ± standard deviation was expressed and compared by Student's *t*-test. When the variables did not conform to a normal distribution, the median of the interquad interval was expressed, and the Wilcoxon rank-sum test was performed. When the variables were classified, they were expressed by quantity and percentage, and they were compared by the chi-squared test and Fisher's exact test.

Principal component analysis was used to classify the measured plasma acylcarnitines and extract factors through factor analysis. The Kaiser–Meyer–Olkin (KMO) value and Bartlett sphericity test are indicators used to test whether the data can be analysed by factor [12]. When the KMO coefficient is greater than 0.5, it indicates that the data can be subjected to factor analysis. Larger KMO coefficients indicate stronger applicability of the data [13]. Varimax rotation was performed on the initial data obtained by factor analysis, and the rotated load matrix was obtained. Rotated data are easier to analyse [14]. The scree plot is a linear graph of the eigenvalues of intuitive response factors; the horizontal axis represents the number of factors, and the vertical axis represents the eigenvalues [15]. An eigenvalue >1 indicates that the cumulative variance contribution rate is ≥60%, and the factor is located on the steep slope of the scree plot as the standard to determine the number of factors.

Model 1 was a univariate binary logistic regression used to estimate odds ratios (OR) and 95% confidence intervals (CI) for acylcarnitine factors in patients with type 2 diabetes. Models 2 to 4 were multivariate binary logistic regressions used to exclude confounding factors. Model 2 adjusted for other acylcarnitines, and Model 3 further adjusted for age, sex, height, weight, BMI, duration of diabetes, HbA1c, and systolic blood pressure (SBP). In addition, Model 4 further adjusted for diabetes medication interference.

## 3. Result

### 3.1. Description of Study Subjects

**Figure 1:**
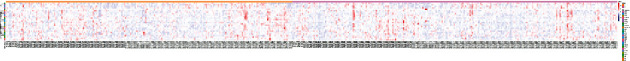
Group A represents the non-DPN group, and Group B represents the DPN group.

All the subjects were T2DM patients, and they had a mean age of 55.88 (±13.16) and a median course of diabetes mellitus of 72 months. There were 275 male and 233 female patients. There were 270 patients with DPN and 238 patients without DPN. Compared to the non-DPN group, T2DM patients with DPN were older, had a longer duration of diabetes, had lower body weight, had small hips, and had lower HbA1c. Patients with diabetic peripheral vascular disease and diabetic retinopathy are more likely to have DPN (Table 1). There were no significant differences in sex, systolic blood pressure (SBP), diastolic blood pressure (DBP), BMI, TG, TC, HDL-C, or LDL-C between the two groups. Compared to non-DPN patients, hydroxylbutyrylcarnitine (C4-OH), succinylcarnitine (C4DC), 3-hydroxyisovalerylcarnitine (C5-OH), octanoylcarnitine (C8), decanoylcarnitine (C10), myristoylcarnitine (C14), 3-hydroxypalmitoylcarnitine (C16-OH), arachidic carnitine (C20), and tetracosanoic carnitine (C24) were lower in DPN patients, but C2 was higher in DPN patients. There was no significant difference in other acylcarnitines between the two groups (Table 2).  Figure 1 represents the trends of all detected metabolites and differential metabolites in the two groups. In the heat map, the colour representing the expression level of related acylcarnitines in the DPN group was darker than that in the non-DPN group (Figure 1).

### 3.2. Extracted Factors of Acylcarnitines

**Figure 2:**
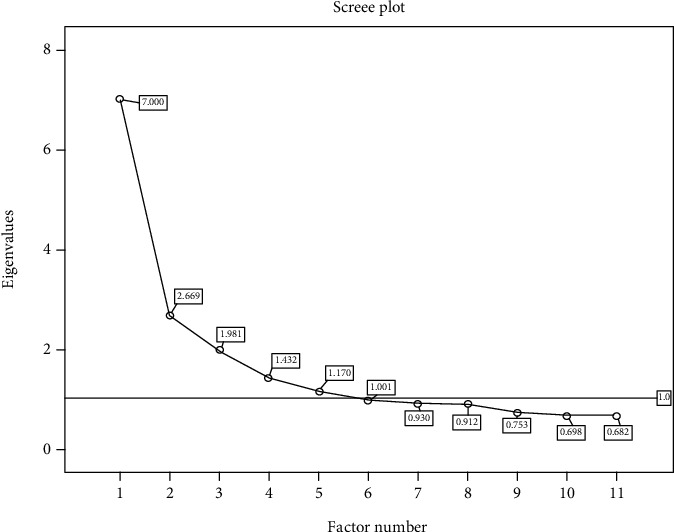
The horizontal axis represents the number of factors, and the vertical axis represents the characteristic value. As shown in the figure, the eigenvalues of Factors 1 to 6 were all greater than 1.

The KMO coefficient of Bartlett's sphericity test was 0.868 (*P* < 0.001), indicating that the factor analysis results were significant. We extracted 6 factors with eigenvalues greater than 1 located on the steep slope of the scree plot (Figure 2), which accounted for 61.02% of the total variance. The rotational loads of 25 acylcarnitines on six factors were listed by the maximal variance method as follows: Factor 1 contained C4DC, C5:1, C14:1, C14DC, C14-OH, C16:1-OH, C20, C22, C24, and C26; Factor 2 contained C2, C3, C4, and C5; Factor 3 contained C8, C10, and C5DC; Factor 4 contained C0, C16, and C18; Factor 5 contained C12, C14, and C16-OH; and Factor 6 contained C5-OH and C6 (Table 3).

### 3.3. Association between Acylcarnitine Extraction Factors and DPN Risk in T2DM

In the univariate analysis, Factor 2 was positively associated with DPN, while Factors 4 and 6 were negatively associated with DPN. The associations persisted after the adjustment for other acylcarnitine factors. After further adjustment for age, height, weight, BMI, SBP, duration of diabetes, HbA1c, and diabetes medication use, the effect of Factor 2 (OR: 1.38, 95% CI: 1.13-1.69) remained largely unchanged (Table 4).

## 4. Discussion

The present study showed that the C2, C3, C4, and C5 short-chain acylcarnitines in Factor 2 were positively correlated with the risk of DPN in T2DM and that this positive correlation existed after adjusting for other confounding factors.

Insulin resistance in muscle and adipose tissue is one of the important causes of T2DM. Following insulin binding to insulin receptors, insulin receptors are phosphorylated, which then activates the Akt cascade and Ras cascade through PI3K and Grb2/Sos [ [16, 17]]. Abnormal insulin signals are the basis of insulin resistance, including insulin receptor degradation, excessive activation of protein tyrosine phosphatase, inflammation, and excessive stress-activated IKKbeta, JNK, ERK, S6K, and mTOR kinase, thereby inhibiting phosphorylation of IRS [18–20]. Similar abnormal insulin signalling also exists in the peripheral nerves of ob/ob mice, including decreased expression of insulin receptors and increased activity of the JNK stress kinase, indicating that DPN is insulin resistant [21]. Insulin resistance may cause mitochondrial dysfunction and ROS increase in nerves [21, 22]. Mitochondrial abnormalities in the nerves of neuropathy patients are usually located in Schwann cells (SCs), which are glial cells that support the survival, function, and repair of peripheral nerve axons after injury [23]. In a mouse model of peripheral neuropathy secondary to mitochondrial dysfunction in SCs, the destruction of SC mitochondria activates a maladaptive integrated stress response through the action of haeme-regulated inhibitor kinase (HRI), which also causes the transformation of lipid metabolism from fatty acid synthesis to oxidation [24]. Studies have shown that SCs rely on the PI3K/AKT/mTOR pathway to conduct insulin/IGF1 signal transduction to promote myelin formation [25]. However, insulin resistance destroys insulin signalling transduction in SCs, depleting important myelin lipid components and eventually leading to demyelination, while acylcarnitine accumulation further aggravates axon loss [25].

Acylcarnitine is an intermediate product of the oxidative catabolism of fatty acids [26]. In cells, fatty acid *β* oxidation is performed in mitochondria, and long-chain fatty acids enter mitochondria under the transport of carnitine [27]. Intracellular long-chain fatty acids are thioesterified by acyl-CoA synthase to form acyl-CoA [27]. Because the mitochondrial intima lacks acyl-CoA transfer proteins, carnitine binds to an activated fatty acid, namely, acyl-CoA, to form acylcarnitine, a process catalysed by carnitine palmityl transferase 1 [28, 29]. Carnitine acylcarnitine transferase is then used to transfer acylcarnitine across the mitochondrial intima, thereby translocating part of the fatty acid conjugated with carnitine to mitochondria [30]. After entering the mitochondria, carnitine is removed from acylcarnitine under the action of carnitine palmityl transferase 2; acyl-coenzyme A is then reformed, and carnitine is returned to the cytoplasm for the next cycle [31]. Mid-chain and short-chain fatty acids enter the mitochondria directly [32]. Acyl-CoA is hydrolysed by a series of enzymes to produce energy [33]. First, acyl-CoA is dehydrogenated by ultralong-chain acyl-CoA dehydrogenase, medium-chain acyl-CoA dehydrogenase, and short-chain acyl-CoA dehydrogenase [33]. Subsequently, the long-chain enoyl-CoA formed by dehydrogenation is dehydrogenated by long-chain enoyl-CoA hydrase for the second time and then catalysed by mitochondrial trifunctional protein (MTP) to cleave the thiolytic enzyme [34]. Medium-chain acyl-CoA and short-chain acyl-CoA perform a similar process through crotonase (short-chain enoyl-CoA hydratase), short-chain (S)-3-hydroxyacyl-CoA dehydrogenase, and medium-chain 3-ketoacyl CoA thiolase [35]. Finally, the resulting acetyl-CoA enters the tricarboxylic acid cycle for energy. In diabetes, mitochondrial substrates are overloaded, and the transport system becomes saturated, resulting in the conversion of acetyl-CoA molecules into acylcarnitine [6]. In a cross-sectional prospective cohort study of 2103 subjects from Beijing and Shanghai, China, who developed T2DM during 6 years of follow-up, plasma concentrations of short-chain, medium-chain, and long-chain acylcarnitines were higher at the baseline, but only long-chain acylcarnitines had a statistically significant correlation with the risk of T2DM [36]. Another study has shown a significant association of short-chain and long-chain acylcarnitines with T2DM risk in the subjects with high cardiovascular risk [37]. Libert et al. found that the ratio of plasma short-chain acylcarnitines to total acylcarnitines is increased in the subjects with metabolic abnormalities, prediabetes, and diabetes combined with obesity compared to metabolically healthy individuals [38]. In addition, a previous study exploring short-chain acylcarnitines and cardiovascular disease (CVD) in 1032 patients with T2DM has reported that elevated C2 levels are associated with an increased risk of CVD in T2DM [39]. A previous study on the prediction of chronic kidney disease progression in patients with T2DM has suggested that C16 is one of the stronger predictors of chronic kidney disease progression in T2DM [40]. Studies on GDM have suggested that plasma acylcarnitines are significantly associated with the risk of GDM, especially in the midterm period, with C4, C8:1, and C16:1OH positively associated with the risk of GDM and C10 and C18 negatively associated with the risk of GDM [41]. These clinical studies show that acylcarnitines have different changes in different stages and complications of diabetes mellitus.

Based on these studies, we hypothesized that insulin resistance leads to mitochondrial dysfunction in SCs of DPN patients and the transition from synthesis to oxidation of fatty acids. Incomplete oxidation of long-chain fatty acids accumulated in the cycle leads to the accumulation of long-chain acylcarnitines and the formation of medium-chain and short-chain acylcarnitines. At the same time, the increased oxidation of long-chain fatty acids inhibits the oxidation of short- and medium-chain fatty acids, leading to an increase in short- and medium-chain acylcarnitines, further damaging peripheral nerves.

There were several limitations in the present study. First, the cross-sectional method used in this study did not allow exploration of the causal relationship between acylcarnitines and DPN in the T2DM population, and hospitalized T2DM cases were selected in this study, which were more severe and did not reflect the overall situation of T2DM. Second, the influence of dietary habits on plasma acylcarnitines was not excluded in this study, but we adjusted for other confounding factors of acylcarnitines, including age, duration of diabetes, BMI, HbA1c, and other demographic and clinical factors, which partially adjusted for the influence of diet. Although the confounding factors were carefully adjusted in this study, unadjusted confounding factors were not excluded.

Our study has important public health implications. As a common complication of T2DM, DPN increases mortality and reduces quality of life in patients with T2DM. As the disease progresses, DPN remains highly prevalent in T2DM even under intensive glucose-lowering therapy. Patients with typical clinical signs and symptoms of DPN are easy to diagnose, while those without obvious signs and symptoms require nerve conduction testing and electrophysiological examination for a definitive diagnosis. In addition, quantitative sensory testing, clinical neurological scales, corneal confocal microscopy, and high-frequency ultrasound are useful in the diagnosis and screening of DPN. Each of these tests has different limitations. For example, their results may be susceptible to subjective factors, and they are invasive, expensive, and time-consuming. Abnormal lipid metabolism is one of the factors that promotes the development of DPN. The present study further explored the changes in plasma acylcarnitine metabolites in patients with DPN to provide new insights into the initial screening and diagnosis of DPN.

## 5. Conclusions

In conclusion, our study showed that C2, C3, C4, and C5 plasma acylcarnitine metabolites of hospitalized Chinese T2DM patients are positively correlated with the risk of DPN. Future cohort and basic studies need to be further explored based on this research.

## Figures and Tables

**Table 1:** Population and clinical characteristics of patients with T2DM.

Variables	DPN	Non-DPN	*P*
N	270 (53.1%)	238 (46.9%)	
Age, years	57.65 ± 11.58	53.86 ± 14.52	0.001∗
Duration of diabetes, month	96.00 (24.00, 168.00)	36.00 (5.50, 120.00)	<0.001∗∗∗
Male, gender	138 (51.1%)	137 (57.6%)	0.145∗∗
Weight, kg	71.08 ± 14.55	74.17(±15.53)	0.012∗
BMI, kg/m^2^	25.10 (22.90, 28.03)	25.39 (23.42, 28.09)	0.105∗∗∗
*BMI categories*			0.110∗∗
<24	104 (38.52%)	70 (29.41%)	
24~ 28	97 (35.93%)	99 (41.60%)	
≥28	67 (24.81%)	65 (27.31%)	
Missing value	2 (0.74%)	4 (1.68%)	
WC, cm	87.84 ± 10.64	89.32 ± 10.87	0.208∗
HC, cm	96.26 ± 7.56	98.65 ± 7.80	0.004∗
WHR, %	0.91 ± 0.08	0.90 ± 0.07	0.340∗
SBP, mmHg	140.96 ± 21.16	138.98 ± 22.10	0.615∗
DBP, mmHg	83.57 ± 11.53	85.58 ± 12.50	0.060∗
CP	1.40 (0.86, 2.00)	1.40 (0.87, 2.12)	0.590∗∗∗
HbA1c, %	9.34 ± 2.27	9.86 ± 2.31	0.029∗
*HbA1c categories*			0.427∗∗
HbA1c ≥ 7	219 (81.1%)	192 (80.7%)	
HbA1c<7	33 (12.2%)	23 (9.7%)	
Missing value	18 (6.7%)	23 (9.7%)	
TG, mmol/L	1.97 (1.18, 3.24)	1.93 (1.23, 3.23)	0.730∗∗∗
TC, mmol/L	5.13 ± 1.28	5.05 ± 1.30	0.539∗
LDL-C, mmol/L	3.09 ± 1.00	3.12 ± 1.05	0.838∗
*LDL-C categories*			0.625∗∗
LDL-C ≥ 2.6	149 (55.2%)	118 (49.6%)	
LDL-C < 2.6	67 (24.8%)	59 (24.8%)	
Missing value	54 (20.0%)	61 (25.6%)	
HDL-C, mmol/L	1.11 ± 0.34	1.15 ± 0.85	0.501∗
*HDL-C categories*			0.337∗∗
<1 in male or < 1.3 in female	129 (47.8%)	92 (38.7%)	
≥1 in male or ≥ 1.3 in female	80 (29.6%)	70 (29.4%)	
Missing value	61 (22.6%)	76 (31.9%)	
DR	40 (14.8%)	19 (8.0%)	0.016∗∗∗
DN	92 (34.1%)	63 (26.6%)	0.068∗∗∗
DPVD	208 (77.0%)	134 (56.3%)	<0.001∗∗∗
Smoking	88 (32.7%)	81 (34.2%)	0.728∗∗∗
Drinking	81 (30.1%)	82 (34.7%)	0.266∗∗∗
*Treatment categories*			<0.001∗∗
Untreated	44 (16.3%)	82 (34.5%)	
Insulin	69 (25.6%)	41 (17.2%)	
Insulin and other antidiabetic medications	59 (21.9%)	36 (15.1%)	
Other antihypertensive drugs	98 (36.3%)	79 (33.2%)	

Data are represented as *n* (%), means ± standard deviation, and median (interquartile range). ^∗^*P* values for comparisons between groups derived by Student's *t*-test. ^∗∗^*P* values for comparisons between groups derived by the chi-squared test. ^∗∗∗^*P* values for comparisons between groups derived by the Wilcoxon signed-rank test.

**Table 2:** Acylcarnitine profile in T2DM patients.

Variables	DPN	Non-DPN	*P*
	Median (interquartile range)	Median (interquartile range)	
C0	25.09 (20.18, 32.43)	25.64 (20.38, 31.80)	0.252
C2	10.63 (7.95, 13.59)	9.65 (7.04, 12.71)	0.012
C3	1.52 (1.12, 2.02)	1.45 (1.03, 2.09)	0.602
C4	0.18 (0.14, 0.23)	0.18 (0.13, 0.24)	0.605
C4-OH	0.04 (0.03, 0.07)	0.05 (0.03, 0.08)	0.001
C4DC	0.29 (0.22, 0.41)	0.33 (0.24, 0.46)	0.006
C5	0.15 (0.11, 0.20)	0.16 (0.12, 0.20)	0.959
C5-OH	0.18 (0.14, 0.24)	0.20 (0.15, 0.28)	0.001
C5DC	0.05 (0.03, 0.09)	0.06 (0.04, 0.09)	0.124
C5:1	0.04 (0.02, 0.05)	0.04 (0.02, 0.06)	0.145
C6	0.07 (0.05, 0.09)	0.07 (0.05, 0.08)	0.289
C8	0.05 (0.04, 0.08)	0.06 (0.04, 0.09)	0.001
C10	0.05 (0.03, 0.07)	0.06 (0.03, 0.09)	0.007
C12	0.05 (0.03, 0.07)	0.05 (0.03, 0.07)	0.543
C14	0.05 (0.04, 0.07)	0.06 (0.04, 0.08)	0.047
C14-OH	0.02 (0.01, 0.04)	0.03 (0.02, 0.04)	0.109
C14DC	0.02 (0.02, 0.04)	0.02 (0.01, 0.04)	0.906
C16	0.73 (0.58, 0.95)	0.72 (0.55, 0.91)	0.437
C16-0H	0.02 (0.01, 0.03)	0.02 (0.02, 0.04)	0.010
C16:1-OH	0.04 (0.03, 0.05)	0.04 (0.03, 0.05)	0.390
C18	0.44 (0.32, 0.55)	0.43 (0.33, 0.59)	0.652
C20	0.02 (0.01, 0.03)	0.02 (0.02, 0.03)	0.004
C22	0.04 (0.03, 0.06)	0.05 (0.03, 0.07)	0.050
C24	0.03 (0.02, 0.05)	0.04 (0.03, 0.05)	0.022
C26	0.03 (0.02, 0.03)	0.03 (0.02, 0.04)	0.589
C14:1	0.05 (0.03, 0.07)	0.05 (0.03, 0.07)	1.000

C0 = free carnitine; C2 = acetylcarnitine; C3 = propionylcarnitine; C4 = butyrylcarnitine; C4-OH = hydroxylbutyrylcarnitine; C4DC =succinylcarnitine; C5 = isovalerylcarnitine; C5-OH = 3-hydroxyisovalerylcarnitine; C5DC = glutarylcarnitine; C5:1 = tiglylcarnitine; C6 = hexanoylcarnitine; C8 = octanoylcarnitine; C10 = decanoylcarnitine; C12 = lauroylcarnitine; C14 = myristoylcarnitine; C14-OH = 3-hydroxyl-tetradecanoylcarnitine; C14DC = tetradecanoyldiacylcarnitine; C14:1 = tetradecenoylcarnitine; C16 = palmitoylcarnitine; C16-OH = 3-hydroxypalmitoylcarnitine; C16 = 1-OH, 3-hydroxypalmitoleylcarnitine; C18 = octadecanoylcarnitine; C20 = arachidic carnitine; C22 = behenic carnitine; C24 = tetracosanoic carnitine; C26 = hexacosanoic.

**Table 3:** Factor analysis of 25 acylcarnitines resulted in factors and the postrotation load of each factor.

Variables	Factor 1	Factor 2	Factor 3	Factor 4	Factor 5	Factor 6
C22	**0.788**	0.153	0.152	-0.028	0.141	-0.095
C26	**0.763**	0.177	-0.007	0.032	-0.057	0.037
C24	**0.716**	-0.040	0.078	0.161	0.008	0.174
C14DC	**0.658**	0.168	0.139	-0.048	0.098	-0.169
C20	**0.653**	-0.155	0.126	0.368	0.054	0.181
C4DC	**0.625**	-0.047	0.040	0.048	0.058	0.353
C16:1-OH	**0.603**	0.238	0.154	0.172	0.150	-0.010
C14:1	**0.543**	0.042	0.531	0.042	0.290	0.068
C5:1	**0.541**	0.100	0.065	-0.029	0.292	0.475
C14-OH	**0.518**	-0.076	0.149	0.118	0.371	0.079
C2	0.034	**0.775**	0.166	0.065	-0.087	-0.133
C3	-0.005	**0.753**	-0.049	0.223	0.041	0.092
C5	0.096	**0.713**	0.044	-0.087	0.013	0.231
C4	0.220	**0.668**	0.176	0.207	0.033	0.083
C8	0.122	0.131	**0.835**	0.132	0.030	0.095
C10	0.087	-0.074	**0.791**	0.328	0.121	0.128
C5DC	0.257	0.244	**0.523**	-0.048	0.120	-0.068
C18	0.222	0.073	0.071	**0.823**	0.086	0.000
C16	0.106	0.444	0.155	**0.664**	0.063	-0.111
C0	0.019	0.282	0.069	**0.497**	0.061	0.404
C12	0.035	0.129	0.045	0.093	**0.756**	0.090
C16-0H	0.249	-0.053	0.074	-0.056	**0.696**	-0.037
C14	0.085	-0.041	0.077	0.407	**0.601**	0.157
C6	0.033	0.235	0.049	-0.092	-0.025	**0.526**
C5-0H	0.502	0.060	-0.058	0.289	0.181	**0.512**

**Table 4:** Acylcarnitines are associated with DPN in univariate and multivariate analyses.

Model	Factor	OR	95% CI	*P*
Model 1	Factor 1	0.91	0.77-1.09	0.309
	Factor 2	1.21	1.01-1.44	0.038
	Factor 3	0.95	0.79-1.13	0.535
	Factor 4	0.83	0.70-1.00	0.046
	Factor 5	1.03	0.87-1.23	0.708
	Factor 6	0.83	0.69-0.99	0.035
Model 2	Factor 1	0.91	0.76-1.09	0.307
	Factor 2	1.21	1.02-1.45	0.036
	Factor 3	0.94	0.78-1.31	0.501
	Factor 4	0.83	0.69-0.99	0.043
	Factor 5	1.04	0.86-1.26	0.670
	Factor 6	0.82	0.69-0.98	0.032
Model 3	Factor 1	0.96	0.79-1.17	0.689
	Factor 2	1.37	1.12-1.67	0.002
	Factor 3	0.94	0.77-1.16	0.582
	Factor 4	0.87	0.71-1.06	0.173
	Factor 5	1.06	0.86-1.30	0.589
	Factor 6	0.87	0.71-1.07	0.179
Model 4	Factor 1	0.97	0.80-1.18	0.754
	Factor 2	1.38	1.13-1.69	0.002
	Factor 3	0.95	0.77-1.16	0.608
	Factor 4	0.86	0.71-1.06	0.152
	Factor 5	1.06	0.86-1.30	0.612
	Factor 6	0.86	0.71-1.06	0.155

Model 1 was a univariate model; Model 2-Model 4 were multivariate models. Among them, Model 2 adjusted the interaction between acylcarnitines. Model 3 further adjusted for the effects of sex, age, height, weight, BMI, SBP, diabetes course, and HbA1c. On this basis, the influence of diabetes drugs was further adjusted to obtain Model 4.

## Data Availability

The data used to support the findings of this study are included within the article.
